# The Relationship Between Affective Visual Mismatch Negativity and Interpersonal Difficulties Across Autism and Schizotypal Traits

**DOI:** 10.3389/fnhum.2022.846961

**Published:** 2022-03-23

**Authors:** Talitha C. Ford, Laila E. Hugrass, Bradley N. Jack

**Affiliations:** ^1^Cognitive Neuroscience Unit, School of Psychology, Deakin University, Geelong, VIC, Australia; ^2^Centre for Human Psychopharmacology, Faculty of Heath, Arts and Design, Swinburne University of Technology, Melbourne, VIC, Australia; ^3^Department of Psychology and Counselling, School of Psychology and Public Health, La Trobe University, Melbourne, VIC, Australia; ^4^Research School of Psychology, The Australian National University, Canberra, ACT, Australia

**Keywords:** electroencephalography, autism, schizotypy, facial emotion processing, visual mismatch negativity

## Abstract

Sensory deficits are a feature of autism and schizophrenia, as well as the upper end of their non-clinical spectra. The mismatch negativity (MMN), an index of pre-attentive auditory processing, is particularly sensitive in detecting such deficits; however, little is known about the relationship between the visual MMN (vMMN) to facial emotions and autism and schizophrenia spectrum symptom domains. We probed the vMMN to happy, sad, and neutral faces in 61 healthy adults (18–40 years, 32 female), and evaluated their degree of autism and schizophrenia spectrum traits using the Autism Spectrum Quotient (AQ) and Schizotypal Personality Questionnaire (SPQ). The vMMN to happy faces was significantly larger than the vMMNs to sad and neutral faces. The vMMN to happy faces was associated with interpersonal difficulties as indexed by AQ Communication and Attention to Detail subscales, and SPQ associated with more interpersonal difficulties. These data suggest that pre-attentive processing of positive affect might be more specific to the interpersonal features associated with autism and schizophrenia. These findings add valuable insights into the growing body of literature investigating symptom-specific neurobiological markers of autism and schizophrenia spectrum conditions.

## Introduction

Autism and schizophrenia share several behavioural and neurobiological characteristics (Lanillos et al., 2020; Poletti and Raballo, 2020; Oliver et al., 2021), particularly pre-attentive processing of visual and auditory information (Jahshan et al., 2012; Randeniya et al., 2018; Thye et al., 2018; Lanillos et al., 2020). The mismatch negativity (MMN) is a well-established event-related potential (ERP) marker of pre-attentive processing of auditory information. The auditory MMN is elicited when an infrequent “deviant” stimulus is randomly presented in a sequence of frequent “standard” stimuli (Naatanen et al., 2007, 2012). There are several theories for the auditory MMN, such as neural fatigue, neural population sharpening, facilitation of stimulus processing, and predictive coding (Naatanen et al., 2007; Wacongne et al., 2012; Stefanics et al., 2014; Kremláček et al., 2016). The most compelling explanation for the auditory MMN is predictive coding, which proposes that the brain generates a probabilistic model of the sensory input (i.e., the standard stimuli) that explains the attenuation of the neural response to the standard over time. The deviant stimulus is a violation of the probabilistic model, and hence results in a larger neural response (Friston, 2005).

Deficits in the auditory MMN have been consistently replicated across the schizophrenia spectrum (Javitt et al., 2000; Umbricht and Krljes, 2005; Naatanen et al., 2011)–from high-risk to first-episode to chronic illness (Javitt et al., 1993; Jahshan et al., 2012; Fulham et al., 2014; Lavoie et al., 2018). Deficits in the auditory MMN in autism are less well established; however, the heterogeneity of autism and inconsistencies in study design is thought to account for this (Schwartz et al., 2018). Nevertheless, recent meta-analyses found reduced auditory MMN in children with autism, but not in adults (Schwartz et al., 2018; Chen et al., 2020). Importantly, the auditory MMN has been associated with poorer social/interpersonal functioning in both autism (Fan and Cheng, 2014) and schizophrenia (Lee et al., 2014; Featherstone et al., 2017), as well as in non-clinical groups (Ford et al., 2017b).

Far fewer studies have investigated whether the visual counterpart of the auditory MMN, the visual MMN (vMMN), is similarly affected in autism and schizophrenia. In schizophrenia studies, a smaller vMMN has been reported in response to infrequent changes on motion direction (Urban et al., 2008), letters (Neuhaus et al., 2013), and sequence of stimulus presentation (Vogel et al., 2018), but not to changes in Gabor patch orientation (Farkas et al., 2015). The vMMN amplitude to motion direction has also been associated with negative symptom severity (Urban et al., 2008). Studies investigating the vMMN in autism have reported reduced vMMN amplitude in adults with autism compared to controls (Cléry et al., 2013b) and longer vMMN latency to object shape deformation in children with autism (Cléry et al., 2013a). No difference in vMMN amplitude or latency was reported for windmill pattern deviants in a small sample of adults (*n* = 11) with autism (Maekawa et al., 2011). One benefit of probing the visual counterpart of the MMN is the ability to utilise social and affective stimuli, such as in the form of faces, to investigate pre-attentive social information processing. Given poor social/interpersonal functioning is a central feature of both autism and schizophrenia, investigating the relationship between pre-attentive social processing and symptom domains could provide valuable insight into the neurobiology of the conditions.

Studies in schizophrenia have reported reduced vMMN to happy and fearful facial expressions (Csukly et al., 2013), as well as neutral facial expressions (Vogel et al., 2018), and the vMMN to happy deviants was associated with better facial emotion recognition (Csukly et al., 2013). Only one study has probed the relationship between facial emotion vMMN and autistic traits in a non-clinical adult population, reporting a reduced vMMN to happy but not sad deviants that was associated with higher overall levels of autistic traits, as quantified using the autism spectrum quotient (AQ; Gayle et al., 2012). These findings, along with those demonstrating that a larger vMMN to happy deviants is associated with better facial emotion recognition, suggest that effective encoding of social information, such as facial emotions, and deviance detection, is necessary for optimal functioning in the social world (Csukly et al., 2013). Investigating the relationship between vMMN to unexpected changes in different types of facial emotions and autism and schizophrenia, which are characterised by social communication difficulties, will further our understanding of the neural mechanisms that are associated with these conditions.

It is well established that many of the symptoms associated with autism and schizophrenia exist on a continuum, from severe clinical pathology to milder traits that present in the non-clinical population. Several studies have utilised non-clinical populations to probe the relationship between trait domains and neurobiological mechanisms (Gayle et al., 2012; Abu-Akel et al., 2017; Donaldson et al., 2017; Ford et al., 2017a,b; Kondo and Lin, 2020), allowing the effects of pharmaceutical interventions and acute psychopathology to be controlled. Furthermore, the relationship between sub-clinical and clinical auditory MMN profiles has been well established, with high-risk individuals and first-degree relatives showing a similar MMN deficit, albeit less pronounced, to clinical groups (Brockhaus-Dumke et al., 2005; Lavoie et al., 2018).

Although there is some evidence to suggest that pre-attentive facial emotion pr ocessing is associated with autism and schizophrenia spectrum symptomatology, the specificity of the vMMN differences to autism and schizophrenia spectrum symptom dimensions remains unknown. This study, therefore, extends that of Gayle et al. (2012) by investigating the extent to which pre-attentive processing of changes in facial emotions are associated with specific autism and schizophrenia spectrum traits in a non-clinical population. It was predicted that a smaller vMMN to happy face deviants would be associated with higher autism and schizophrenia trait severity, particularly in the social domain. The extent to which vMMN to happy and sad face deviants predict the positive and disorganised schizotypy dimensions and attention and imagination dimensions of autism were explored.

## Materials and Methods

Ethical approval for this study was granted by the Swinburne University Human Research Ethics Committee (2016/092) in accordance with the Declaration of Helsinki. All participants provided written informed consent prior to commencing the study.

### Participants

A total of 77 non-clinical adults aged 18–40 years participated in this study. All participants had normal or corrected to normal vision, and were free from psychotropic medications and psychiatric illness, except for one male participant taking an SSRI for mild depression who was excluded from analyses. One male participant was removed due to incomplete psychometric data, and an additional 15 were excluded due to poor electroencephalography (EEG) data (9 female, 6 male), resulting in 61 participants in the final sample (32 female, 29 male). See Table 1 for final sample participant characteristics, including Mann-Whitney U non-parametric tests to identify sex differences in continuous variables, and χ2 to identify sex differences in self-reported handedness. Males scored slightly higher than females on AQ Attention Switching and Schizotypal Personality Questionnaire (SPQ) Disorganisation dimensions (*p* < 0.05).

**TABLE 1:** Final sample characteristics for full-scale autism spectrum quotient and schizotypal personality questionnaire total and dimension scores.

	Total Mean(SE)	Range Min-Max	Female Mean(SE)	Male Mean(SE)	Mann-Whitney *U*	*p*-value
N	61		32	29		
Age	25.2(0.62)	18–37	24.9(0.84)	25.6(0.92)	435.0	0.680
AQ total	105.0(2.90)	63–171	99.0(3.84)	111.5(4.12)	328.0	0.050
Social skills	20.0(0.82)	10–37	19.2(1.04)	21.0(1.27)	382.0	0.238
Communication	19.1(0.76)	10–35	17.7(0.99)	20.7(1.12)	335.5	0.064
Attention switching	23.2(0.66)	11–34	21.3(0.91)	25.3(0.81)	260.5	0.003*
Attention to detail	23.2(0.71)	13–35	22.4(1.06)	24.2(0.93)	366.0	0.158
Imagination	19.4(0.67)	11–36	18.4(0.75)	20.4(1.13)	401.5	0.369
SPQ total	146.3(4.88)	84–231	136.8(6.29)	156.9(7.18)	329.0	0.051
Cognitive-perceptual	57.3(2.90)	33–102	52.9(2.99)	62.3(3.71)	341.0	0.077
Interpersonal	54.3(1.91)	31–93	52.3(2.39)	56.6(3.03)	406.5	0.410
Disorganisation	34.7(1.41)	16–60	31.5(1.68)	38.1(2.16)	316.0	0.033*
Handedness (R:L)	54:7		26:6	28:1	χ2,3.51	0.061

*SE, standard error; AQ, autism spectrum quotient; SPQ, schizotypal personality questionnaire; χ^2^, chi square test.*

*p < 0.05 corrected.

### Measures

The full 50-item AQ was used to measure the five autism trait dimensions of Social Skill, Communication, Attention Switching, Attention to Detail and Imagination (Baron-Cohen et al., 2001). The 74-item SPQ was used to measure schizophrenia domains of Cognitive-Perceptual Features, Interpersonal Features and Disorganised Features (Raine, 1991). AQ and SPQ items were pseudo-randomised and presented on a 4-point Likert scale from 1 (*strongly disagree*) to 4 (*strongly agree*) in order to improve reliability (Wuthrich and Bates, 2005; Ford and Crewther, 2014). All statistical analyses were conducted using the full-scale scoring system, which retained 1–4 scores for each item. We also applied the traditional scaling system to the AQ and SPQ items such that *strongly agreee* and *agree* = 1, and *strongly disagree* and *disagree* = 0, which is in line with Gayle et al. (2012), and present all relevant anayses in the (Supplementary Tables 1, 2, 5). Participants trait depression, anxiety and stress scores were collected using the DASS-21 (Lovibond and Lovibond, 1995), in order to control for these effects on the relationships between AQ and SPQ domains and the vMMN.

### Stimuli

Face stimuli were selected from the Nimstim database of standardised facial expressions (Tottenham et al., 2009). Five grey-scale female faces were selected; we used female faces only to remove gender bias toward faces. All faces were of caucasian appearance, and the happy, sad and neutral emotions for each face was included. We used the SHINE toolbox for Matlab to match faces for mean luminance (Willenbockel et al., 2010).

### Procedure

Participants were seated 57 cm from the stimulus presentation computer screen (inside an electrically shielded room). Participants were instructed to focus on a fixation cross (i.e., “+”) in the centre of the screen that randomly alternated in colour between red and green, and to respond as quickly as possible *via* keypress when the cross changed colour. The face stimuli for the vMMN task were presented directly behind the fixation cross during the colour-change distractor task.

**FIGURE 1:**
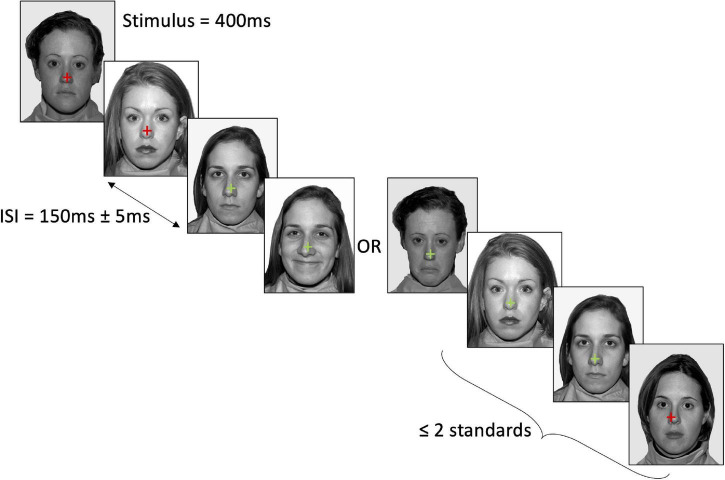
Example stimulus presentation in the neutral standard–happy deviant–sad deviant condition. Stimuli were presented for 400 ms with a 150 ± 5 ms jitter. At least two standard faces were presented between each deviant. Participants attended to the cross in the centre of the face and pressed the relevant key when the cross changed colour. Face stimuli were sourced from the freely available Nimstim database (Tottenham et al., 2009).

The vMMN task consisted of 6 blocks containing a total of 750 trials (600 standards, 75 trials for both deviant emotions), with each facial emotion serving as standard and deviant across the blocks (e.g., happy standard, neutral and sad deviant; neutral standard, happy and sad deviant; sad standard, happy and neutral deviant). Each face identity was presented an equal number of times in each block (i.e., 120 presentations of each face as standard emotion, and 15 presentations of each face as deviant emotion). Block order was counterbalanced across participants using a randomised balanced Latin-square. Face stimulus presentation was pseudo-randomised, ensuring that the same identity was not shown on consecutive trials, and that standard emotions were presented for the first three and final two trials of each block, and at least two standard emotions were presented between each deviant emotion. Face stimuli were presented for 400 ms, with an inter-stimulus interval of 150 ms and a jitter of ± 50 ms. The participant’s task was to attend to a fixation cross in the centre of the face stimuli, and to press the left shift key with their left hand when the colour of the fixation cross changed from red to green, or the right shift key with their right hand when the colour changed from green to red (see Figure 1). Changes in the fixation colour occurred at randomised time intervals (*M* = 5.50 s, SD = 3.03 s) that were not correlated with the presentation order of the facial emotions. This task ensured that participants were looking at the face stimuli, but were not attending to it. Analyses of the key press data confirmed that the distractor task was performed to a satisfactory level throughout the vMMN recording, with all participants responding within 2 s on at least 80% of trials (*M* = 97.93%, SD = 2.75%; MRT = 650 ms, SDRT = 99 ms).

### Electroencephalography Acquisition

Electroencephalography was recorded using a 64-channel Quickcap (Neuroscan, Compumedics). Electrode site Fz served as ground and linked mastoid electrodes were used as a reference. The data were bandpass filtered online (0.1–200 Hz) and sampled at 1 kHz. To monitor blinks, vertical electro-oculargraphy (EOG) was recorded from electrodes above and below the right eye.

### Electroencephalography Pre-processing

The EEG data were pre-processed in Brain Vision Analyser, Version 2.1 (Brain Products GmBH, Germany). We re-referenced the data offline to the average of all scalp electrodes, and filtered the data using a half-amplitude 0.1 to 30 Hz phase-shift free Butterworth filter (24 dB/Oct slope). EEG data were epoched from −100 to 500 ms in relation to face stimulus onset, and epochs were baseline-corrected to their mean voltage from −100 to 0 ms. Epochs with signals exceeding peak-to-peak amplitudes of 100 microvolts at EEG or EOG channel were excluded. This resulted in the rejection of 14% of all trials. All standard trials immediately following a deviant were excluded from analyses.

Event-related potentials for each emotion (happy, neutral, sad) and for each context (standard, deviant) were computed from occipital electrodes (PO7, O1, Oz, O2, PO8). The time-window for the vMMN was identified using a collapsed localiser approach (Luck and Gaspelin, 2017). All standards and deviants were collapsed across all electrodes and facial emotions, and a difference wave (vMMN) was calculated by subtracting the collapsed standards from the collapsed deviants. The vMMN was then identified as the negative peak between 150 and 300 ms, which occurred at 237 ms. The vMMN time-window was then determined as the 100 ms around the vMMN peak (i.e., 187–287 ms). This time-window was used to analyse the uncollapsed vMMNs at each electrode, which were calculated by subtracting the mean standard waveform from the mean deviant waveform for each facial emotion (i.e., happy vMMN = happy deviant–happy standard).

### Statistical Analysis

An *a priori* power analysis conducted using the *pwr* package in R (Champely, 2018) indicated that 63 participants were required to detect a moderate effect (*r* = 0.343, based on Gayle et al., 2012) with 80% power using correlations with alpha = 0.05.

A 3 (laterality: left [PO7, O1], central [Oz], right [PO8, O2]) × 3 (emotion: happy, sad, neutral) linear mixed effects analysis was conducted to investigate vMMN differences across laterality and emotion, with subject entered as a random factor. To investigate the extent to which the happy, sad, and neutral vMMN at different sites was related to autism and schizophrenia trait domains, non-parametric Spearman rank order correlations were calculated. We calculated non-parametric correlation coefficients due to significantly skewed distributions of most trait domains and vMMN values (Shapiro-Wilk *p* < 0.05). Age and trait depression, anxiety, and stress were not related to the happy or sad vMMN at left, central, and right sites, thus were not added as covariates in the analyses. Mann-Whitney *U*-tests probed sex differences in AQ and SPQ total and dimension scores (see Table 1). Given the significant sex difference in AQ Attention Switching, we ran partial correlations controlling for sex for the relationship between AQ Attention Switching and the vMMN for happy, sad, and neutral faces at each site. There were no significant correlations found (*p*s > 0.05; see Supplementary Table 3). We also probed electrode-specific relationships between the vMMN and AQ and SPQ subscales, which are reported in Supplementary Tables 4, 5. Family wise error was corrected for by applying false discovery rate FDR; (Benjamini and Hochberg, 1995). Statistical analyses were conducted using the *psych*, *lme4*, and *Hmisc* packages in R (Bates et al., 2015; Revelle, 2018; Harrell, 2019).

## Results

**FIGURE 2:**
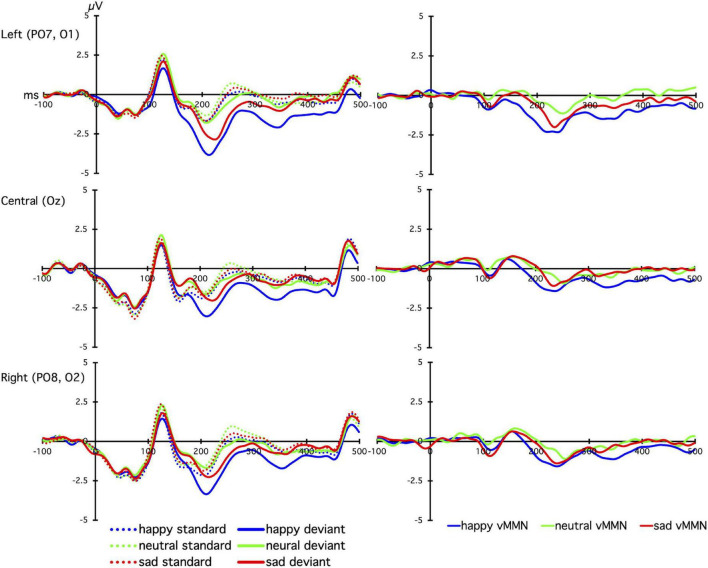
The line graphs show the grand-averaged standard (dashed lines) and deviant (solid lines) event related potentials (left panel), and deviant-minus-standard difference waves (visual mismatch negativity; vMMN, right panel), for happy (blue), neutral (green), and sad (red) facial expressions. Plots show time (milliseconds; ms) on the *x*-axis, with 0 indicating stimulus onset of the emotional face, and voltage (microvolts; μV) on the *y*-axis, with positive voltages plotted upward.

**FIGURE 3:**
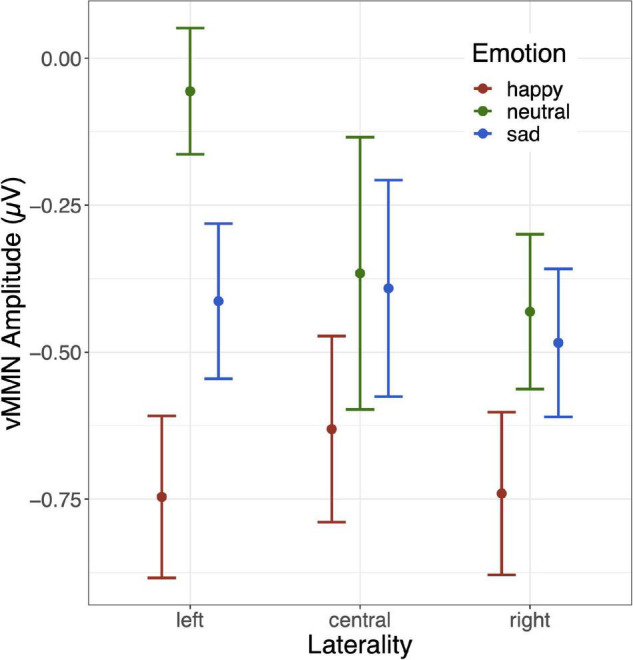
Mean and standard error bars for the 3 (laterality: left [PO7, O1], central [Oz], right [O2, PO8]) × 3 (emotion: happy, sad, neutral) linear mixed effects model.

The grand mean average vMMNs for each facial emotion over each site are shown in Figure 2, and the laterality × emotion linear mixed effects plot of mean vMMN is shown in Figure 3. The linear mixed effects analysis (laterality × emotion) revealed a significant main effect for emotion, *F*_(2, 826)_ = 6.89, *p* = 0.001, with the happy vMMN larger than the neutral vMMN, *t*(587.79) = −3.84, *p* < 0.001, and sad vMMN, *t*(587.43) = −2.42, *p* = 0.016. There was no difference between the sad and neutral vMMNs, t(587.91) = −1.46, *p* = 0.145. No significant laterality main effect or emotion x laterality interactions were found (*p*s < 0.1; Figure 3).

**FIGURE 4:**
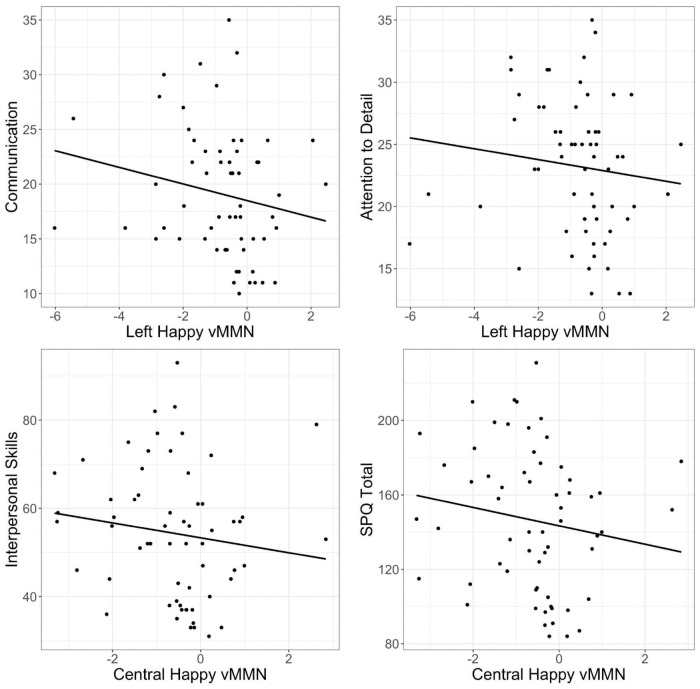
Scatter plots of correlations between left and central visual mismatch negativity (vMMN) amplitude (microvolts; μV) to happy faces (happy deviant–happy standard) and Communication, Attention to Detail, and Interpersonal subscales, and Schizotypal Personality Questionnaire (SPQ) total.

Spearman rank order correlations investigating the relationship between the vMMN to happy, sad, and neutral facial expressions found significant moderate negative correlations between the happy vMMN at left and central sites and social autism and schizophrenia spectrum domains (*p*s < 0.05; see Table 2). This indicates that those with more social and communication difficulties and higher attention to detail exhibit a larger (more negative) vMMN over the left and central sites (Figure 4); however, these correlations did not survive correction for FDR. There were no significant relationships between sad and neutral vMMN amplitudes at left, central, or right sites for any of the AQ or SPQ domains (*p*s > 0.05).

**TABLE 2:** Spearman rank order correlations between full-scale autism spectrum quotient and schizotypal personality questionnaire dimension scores and average visual mismatch negativity amplitudes across occipital electrode sites for each emotional expression.

	Happy vMMN			Sad vMMN			Neutral vMMN		
	Left	Central	Right	Left	Central	Right	Left	Central	Right
Social skills	−0.18	−0.18	−0.06	0.13	0.0	0.02	−0.18	−0.09	−0.04
Communication	−0.29*	−0.23	−0.13	0.19	0.12	0.1	−0.1	−0.11	−0.01
Attention switching	−0.1	−0.22	−0.08	0.16	0.09	0.2	−0.11	−0.12	−0.06
Attention to detail	−0.25*	−0.14	−0.15	−0.01	0.04	0.02	0.01	0.08	0.14
Imagination	−0.05	−0.03	0.12	0.05	0.02	0.05	−0.23	−0.14	−0.22
AQ total	−0.23	−0.2	−0.09	0.12	0.07	0.07	−0.17	−0.1	−0.04
Cognitive-perceptual	−0.18	−0.2	0.03	0.09	0.06	0.13	−0.05	−0.07	−0.01
Interpersonal	−0.24	−0.25*	−0.1	0.13	0.03	0.0	−0.1	−0.02	0.02
Disorganised	−0.1	−0.18	−0.08	0.19	0.07	0.08	−0.02	−0.05	−0.03
SPQ total	−0.22	−0.27*	−0.09	0.13	0.04	0.08	−0.05	−0.04	0.01

*vMMN, visual mismatch negativity; AQ, autism spectrum quotient; SPQ, schizotypal personality questionnaire.*

*p < 0.05.

*No correlations survived correction for false discovery rate.*

In order to compare the current data with previous results (Gayle et al., 2012), we re-ran the AQ and SPQ correlations with the vMMN calculated for happy and sad deviants relative to the neutral standards (Table 3). Similar to the original analyses (i.e., when the vMMN was calculated for the same emotion when it was deviant relative to when it was the standard; see Table 2), the communication domain of AQ and the total SPQ score were negatively correlated with the vMMN, however, these correlations were weak and not significant (*p*s < 0.05).

**TABLE 3:** Spearman rank order correlations between full-scale autism spectrum quotient and schizotypal personality questionnaire dimension scores and average visual mismatch negativity amplitudes when the mismatch response was calculated for happy and sad deviants relative to neutral standards.

	Happy vMMN			Sad vMMN		
	Left	Central	Right	Left	Central	Right
Social skills	–0.16	–0.1	–0.24	0.07	–0.03	–0.05
Communication	–0.15	–0.16	–0.20	0.07	0.05	–0.04
Attention switching	0.03	0.06	0.01	0.07	0.06	0.1
Attention to detail	–0.11	–0.14	–0.05	–0.01	0.09	–0.12
Imagination	0.03	0.16	0.11	–0.02	–0.03	–0.05
AQ total	–0.10	–0.05	–0.11	0.05	0.03	–0.06
Cognitive-perceptual	–0.16	–0.21	–0.06	–0.04	–0.06	–0.15
Interpersonal	–0.14	–0.13	–0.16	–0.02	0.02	–0.18
Disorganised	0.04	–0.09	–0.02	–0.01	–0.03	–0.19
SPQ total	–0.15	–0.21	–0.12	–0.04	–0.02	–0.21

*vMMN, visual mismatch negativity; AQ, autism spectrum quotient; SPQ, schizotypal personality questionnaire.*

## Discussion

This study is the first to investigate the relationship between emotion-related vMMN amplitudes and specific autism and schizophrenia spectrum traits within the non-clinical population. This work extends from Gayle et al. (2012) who reported a moderate association between the happy vMMN and total AQ scores at PO8, but did not explore specific autism trait domains, or the schizophrenia spectrum. As expected, there were strong bilateral occipitotemporal vMMN responses to the happy and sad facial expressions. Although vMMN amplitudes were not significantly correlated with the total AQ score in this study, we found that people with higher levels of interpersonal difficulty specifically (as indexed by the AQ Communication and Attention to Detail, and SPQ Interpersonal Features subscales) tended to exhibit stronger vMMN responses to happy expressions. Given the number of statistical tests conducted, however, none of these correlations survived correction for FDR. Nevertheless, this well-designed study revealed associations, although moderate, that were in the opposite direction to those previously reported (Gayle et al., 2012), and hence is an important Contribution To The Field.

These data reveal a moderate relationship between interpersonal difficulties and the left and central vMMN to happy faces, such that vMMN was larger with more interpersonal difficulties, which was in the opposite direction to Gayle et al. (2012). Gayle et al. (2012) only reported the association between the right lateral (PO8) happy vMMN and AQ, and a notable methodological difference is that they calculated the vMMN responses to happy and sad deviants relative to neutral standards. Hence, differences in the morphology of the neural response to the standard and deviant might reflect differences in response to the specific stimulus being presented (i.e., a sad face vs. a neutral face), as opposed to a neural response to a prediction violation. Gayle et al.’s (2012) results may have reflected a correlation between AQ and the evoked responses to facial emotion, rather than a correlation between AQ and responses to emotion prediction error. However, our subsequent analyses showed that regardless of whether we compared deviants and standards for the same emotion (i.e., happy deviant vs. happy standard), or compared emotional deviants with neutral standards, correlations between the happy vMMN amplitude and autistic and schizotypal personality traits tended to be negative (see Supplementary Tables 4, 5). Thus, our results indicate that predictive error responses to unexpected happy faces tend to be stronger for people with higher levels of interpersonal difficulties, as indexed by the subscales of the AQ and SPQ.

Although the increase in vMMN to happy deviants (compared to happy standards) with more social and interpersonal difficulties was unexpected, previous studies of the auditory MMN in Asperger’s Syndrome and autism have also reported conflicting findings. For instance, Lepistö et al. (2007) found that adults with Asperger’s Syndrome tended to exhibit enhanced MMNs to speech sounds, but not for non-speech sounds, suggesting an auditory hypersensitivity or filtering difficulty in Asperger’s Syndrome (Lepistö et al., 2007). Furthermore, Schwartz et al. (2018) meta-analysis found that while the auditory MMN was generally reduced in children with autism, adults with autism elicit a larger MMN to speech deviants compared to controls. However, they highlighted that many studies of the auditory MMN had small sample sizes and did not properly counterbalance their stimuli.

We observed moderate relationships between autistic and schizotypal traits and emotion-related vMMN amplitudes for happy facial expressions (uncorrected), but not for sad or neutral expressions. This finding is consistent with Gayle et al. (2012) who reported that AQ scores were selectively related to vMMN in response to happy expressions, but are surprising in relation to evidence that people with autism exhibit more general deficits in affective processing (Wright et al., 2008; Fan and Cheng, 2014; Eack et al., 2015). It could be that different aspects of affective processing have different relationships with autistic traits; for instance, Wilbarger et al. (2009) found that people with autism exhibit an atypical startle response to positively valanced faces, despite showing typical implicit responses with automatic facial mimicry and overt valence ratings.

The vMMN to happy faces over the left sites were also shown to increase moderately with more AQ Attention to Detail (uncorrected), which is somewhat intuitive given better preattentive discrimination between emotions could lend itself to more superior attention to detail in the social environment. Again, better discrimination of happy deviants among neutral and sad standard faces may not necessarily translate to correct interpretation of facial expressions. Finally, SPQ scores were associated with larger vMMN amplitudes for the happy facial expressions (uncorrected). This effect was specific to the central occipital region (Oz) and was driven by weak-moderate relationships across the three schizotypy dimensions (Cognitive-Perceptual, Interpersonal and Disorganised Features).

Our design allowed the vMMN to be computed as the difference between the deviant and standard of each emotion and removed a particular potential bias toward happy and sad emotion compared to the neutral emotion. A further strength was the use of greyscale faces of five different female actors. The use of female-only stimuli and equal number of male and female participants recruited in this study removed any potential sex differences in the vMMN (Kecskes-Kovacs et al., 2013), and allowed us to be more confident that the vMMN was due to emotion prediction error, rather than a sex prediction error. Furthermore, through investigating the relationship between the vMMN to facial emotions and varying degrees of specific autism and schizophrenia spectrum symptom traits in a non-clinical sample, these findings highlight the utility of this population to better understand the neurobiology of symptoms at a clinical level. Indeed, the relationship between sub-clinical and clinical auditory MMN profiles has been well established, with high-risk individuals and first-degree relatives showing a similar MMN deficit, albeit less pronounced, to clinical groups (Brockhaus-Dumke et al., 2005; Lavoie et al., 2018).

Despite the robust research methods and large sample size employed herein, there are some limitations that should be taken into consideration when interpreting these data. First, we did not include a non-emotional deviant, such as stimulus tint. Instead, we calculated the vMMN by subtracting the deviant of each emotion from the standard of the same emotion (i.e., happy deviant minus happy standard), which ensured the detected vMMN was not simply a response to the physical characteristics of faces or to the change in emotional expression. Furthermore, although participants were excluded if they were taking psychoactive medications, they were not asked to abstain from caffeine, nicotine, alcohol, cannabis, or other illicit substances prior to testing. Only moderate alcohol consumption (0.04–0.07% BAC) has been shown to affect the vMMN (Kenemans et al., 2010), but acute doses of nicotine, alcohol and cannabis affect the auditory MMN, an thus may also effect the visual counterpart. Given testing for this study occurred on weekdays, it is likely that participants were only acutely affected by caffeine, which has been shown not to affect auditory MMN (Rosburg et al., 2004). Nevertheless, these data should be interpreted with the above limitations in mind. Finally, due to the large number of statistical tests performed, none of the correlations survived correction for FDR. Future research should be conducted on a larger sample, or with more targetted hypotheses, to replicate these findings.

This study sought to further explore the findings of Gayle et al. (2012) by investigating the relationship between the vMMN to happy and sad facial expressions and specific autism and schizophrenia spectrum traits in a large sample of non-clinical adults. We addressed the research design limitations outlined in previous studies and found that, contrary to expectations, communication and interpersonal difficulties, as well as attention to detail, were associated with a greater vMMN amplitude in response to happy faces; however, the statistical significance of these effects diminished following correction for FDR. These contrary findings are a valuable contribution to the growing body of literature investigating symptom-specific neurobiological markers of autism and schizophrenia spectrum conditions.

## Data Availability Statement

The raw data supporting the conclusions of this article will be made available by the authors, without undue reservation.

## Ethics Statement

The studies involving human participants were reviewed and approved by Swinburne University Human Research Ethics Committee. The patients/participants provided their written informed consent to participate in this study.

## Author Contributions

TF, LH, and BJ conceptualised the project and developed the study materials, reviewed, and edited manuscript drafts. TF and LH conducted participant recruitment and data collection, and drafted the manuscript. BJ analysed the EEG data. TF conducted statistical analyses. All authors contributed to the article and approved the submitted version.

## Conflict of Interest

The authors declare that the research was conducted in the absence of any commercial or financial relationships that could be construed as a potential conflict of interest.

## Publisher’s Note

All claims expressed in this article are solely those of the authors and do not necessarily represent those of their affiliated organizations, or those of the publisher, the editors and the reviewers. Any product that may be evaluated in this article, or claim that may be made by its manufacturer, is not guaranteed or endorsed by the publisher.
