# Supplementary material for: The Relationship Between Affective Visual Mismatch Negativity and Interpersonal Difficulties Across Autism and Schizotypal Traits
Source: Front Hum Neurosci. 2022 Mar 23;16:846961. doi: 10.3389/fnhum.2022.846961 (PMC8983815; doi:10.3389/fnhum.2022.846961)
Supplement: Supplementary file 1 [file Data_Sheet_1.pdf]

Supplementary Material

The relationship between affective visual mismatch negativity and interpersonal difficulties across autism and schizotypal traits

Talitha C. Ford, Laila E. Hugrass, Bradley N. Jack

Supplementary Table 1. Sample characteristics for Scaled Autism Spectrum Quotient and Schizotypal Personality Questionnaire total and dimension scores

	Total Mean(*SE*)	Female Mean(*SE*)	Male Mean(*SE*)	Mann-Whitney *U*	*p*-value
N	61	32	29		
AQ total	16.73(1.17)	14.50(1.56)	19.21(1.67)	332.0	0.057
Social Skills	2.83(0.36)	2.56(0.44)	3.14(0.57)	423.0	0.554
Communication	2.54(0.3)	2.03(0.39)	3.10(0.45)	348.0	0.089
Attention Switching	4.23(0.31)	3.43(0.43)	5.10(0.39)	273.5	0.006*
Attention to Detail	4.47(0.32)	4.09(0.47)	4.90(0.42)	377.0	0.207
Imagination	2.66(0.26)	2.38(0.29)	2.97(0.45)	419.0	0.514
SPQ total	23.61(1.94)	19.31(2.42)	28.34(2.89)	313.5	0.030*
Cognitive-Perceptual	7.69(0.95)	5.88(1.13)	9.69(1.49)	328.5	0.050
Interpersonal skills	9.67(0.78)	8.63(0.99)	10.83(1.21)	377.5	0.213
Disorganisation	6.25(0.62)	4.81(0.74)	7.83(0.94)	302.0	0.019*

AQ = Autism Spectrum Quotient, SPQ = Schizotypal Personality Questionnaire, * p <. 05 *uncorrected*

Supplementary Table 2. Spearman rank order correlations between Scaled Autism Spectrum Quotient and Schizotypal Personality Questionnaire dimensions and average visual mismatch negativity amplitudes across occipital electrode sites for each emotional expression.

	Happy vMMN			Sad vMMN			Neutral vMMN		
	left	central	right	left	central	right	left	central	right
Social skills	-0.17	-0.24	-0.13	0.1	0.02	-0.05	-0.18	-0.09	-0.04
Communication	-0.25	-0.22	-0.18	0.14	0.11	0.04	-0.01	-0.04	0.07
Attention Switching	-0.07	-0.22	-0.12	0.15	0.11	0.16	-0.08	-0.11	-0.01
Attention to Detail	-0.31*	-0.22	-0.23	0.06	0.1	0.09	0.01	0.1	0.16
Imagination	-0.08	-0.13	-0.05	0.05	0	0.03	-0.14	-0.07	-0.1
AQ total	-0.23	-0.26*	-0.19	0.11	0.06	0.05	-0.12	-0.08	0.01
Cognitive-Perceptual	-0.17	-0.23	-0.01	0.1	0.04	0.1	-0.05	-0.06	0
Interpersonal	-0.24	-0.28*	-0.13	0.07	-0.01	-0.06	-0.04	0	0.04
Disorganised	-0.12	-0.2	-0.15	0.2	0.07	0.06	-0.01	-0.04	0
SPQ total	-0.22	-0.29	-0.13	0.11	0.01	0.03	-0.03	-0.03	0.01

Notes: vMMN = visual mismatch negativity, AQ = Autism Spectrum Quotient, SPQ = Schizotypal Personality Questionnaire. *$p < .05$. No correlations survived correction for false discovery rate.

Supplementary Table 3. Partial Spearman rank order correlations between full-scale Autism Spectrum Quotient Attention Switching and average visual mismatch negativity amplitudes across visual electrode sites for each emotional expression, controlling for sex.

	Happy vMMN			Sad vMMN			Neutral vMMN		
	left	central	right	left	central	right	left	central	right

Attention switching	-0.04	-0.18	-0.06	0.14	0.08	0.12	-0.15	-0.20	-0.11

Notes: vMMN = visual mismatch negativity

Supplementary Table 4. Spearman rank order correlations between Full-Scale Autism Spectrum Quotient and Schizotypal Personality Questionnaire dimensions and average visual mismatch negativity amplitudes across individual electrode sites for each emotional expression.

	Happy vMMN					Sad vMMN					Neutral vMMN				
	PO7	O1	Oz	O2	PO8	PO7	O1	Oz	O2	PO8	PO7	O1	Oz	O2	PO8
Social skills	-0.08	-0.22	-0.18	-0.17	-0.05	0.14	0.11	0.0	-0.01	0.0	-0.17	-0.11	-0.09	-0.13	0.04
Communication	-0.21	-0.28*	-0.23	-0.27*	-0.07	0.21	0.15	0.12	0.06	0.1	-0.09	-0.07	-0.11	-0.08	0.05
Attention Switching	-0.09	-0.09	-0.22	-0.16	-0.12	0.13	0.17	0.09	0.17	0.17	-0.06	-0.07	-0.12	-0.09	-0.07
Attention to Detail	-0.2	-0.27*	-0.14	-0.28*	-0.08	0.01	0.0	0.04	0.03	-0.03	0.04	0.03	0.08	0.03	0.14
Imagination	0.03	-0.11	-0.03	0.0	0.15	0.01	0.04	0.02	-0.02	0.05	-0.24	-0.18	-0.14	-0.22	-0.16
AQ total	-0.14	-0.26*	-0.2	-0.22	-0.06	0.12	0.12	0.07	0.03	0.04	-0.15	-0.13	-0.1	-0.14	0.01
Cognitive-Perceptual	-0.16	-0.15	-0.2	-0.17	0.12	0.14	0.04	0.06	0.08	0.13	-0.07	-0.04	-0.07	-0.12	0.03
Interpersonal	-0.14	-0.28*	-0.25*	-0.24	-0.01	0.14	0.13	0.03	-0.02	-0.01	-0.06	-0.06	-0.02	-0.13	0.11
Disorganised	-0.12	-0.06	-0.18	-0.27*	0.06	0.22	0.11	0.07	0.02	0.09	0.03	-0.03	-0.05	-0.08	-0.01
SPQ total	-0.19	-0.2	-0.27*	-0.29*	0.04	0.17	0.08	0.04	0.03	0.09	-0.02	-0.04	-0.04	-0.1	0.05

Notes: vMMN = visual mismatch negativity, AQ = Autism Spectrum Quotient, SPQ = Schizotypal Personality Questionnaire. *$p < .05$ *uncorrected*. No correlations survived correction for false discovery rate.

Supplementary Table 5. Spearman rank order correlations between Scaled Autism Spectrum Quotient and Schizotypal Personality Questionnaire dimensions and average visual mismatch negativity amplitudes across individual electrode sites for each emotional expression.

	Happy vMMN					Sad vMMN					Neutral vMMN				
	PO7	O1	Oz	O2	PO8	PO7	O1	Oz	O2	PO8	PO7	O1	Oz	O2	PO8
Social skills	-0.06	-0.22	-0.24	-0.2	-0.1	0.13	0.09	0.02	-0.06	-0.05	-0.17	-0.14	-0.09	-0.1	0.03
Communication	-0.20	-0.23	-0.22	-0.3	-0.1	0.18	0.1	0.11	0.02	0.02	0.0	0.0	-0.04	0.02	0.12
Attention Switching	-0.07	-0.04	-0.22	-0.17	-0.17	0.11	0.18	0.11	0.14	0.13	-0.02	-0.04	-0.11	-0.02	-0.05
Attention to Detail	-0.28*	-0.31*	-0.22	-0.32*	-0.17	0.08	0.07	0.1	0.08	0.05	0.03	0.03	0.1	0.05	0.19
Imagination	-0.05	-0.10	-0.13	-0.16	0.05	0	0.07	0	-0.07	0.04	-0.11	-0.13	-0.07	-0.09	-0.08
AQ total	-0.17	-0.24	-0.26*	-0.31	-0.14	0.13	0.1	0.06	0.01	0.02	-0.07	-0.09	-0.08	-0.06	0.06
Cognitive-Perceptual	-0.18	-0.13	-0.23	-0.21	0.09	0.16	0.03	0.04	0.05	0.1	-0.05	-0.04	-0.06	-0.12	0.05
Interpersonal	-0.13	-0.26*	-0.28*	-0.24	-0.06	0.09	0.08	-0.01	-0.07	-0.07	0.01	-0.02	0.0	-0.11	0.12
Disorganised	-0.15	-0.07	-0.2	-0.31*	0.0	0.24	0.11	0.07	0.01	0.08	0.07	-0.02	-0.04	-0.04	0.02
SPQ total	-0.2	-0.19	-0.29*	-0.31*	0.0	0.16	0.05	0.01	-0.01	0.04	0.02	-0.02	-0.03	-0.1	0.06

Notes: vMMN = visual mismatch negativity, AQ = Autism Spectrum Quotient, SPQ = Schizotypal Personality Questionnaire. *$p < .05$ *uncorrected*. No correlations survived correction for false discovery rate.
